# 工作环境烟草暴露与肺癌发生危险的系统评价

**DOI:** 10.3779/j.issn.1009-3419.2011.04.08

**Published:** 2011-04-20

**Authors:** 新卓 王, 玉坤 秦, 俊东 谷, 凤玮 王, 培杰 贾, 辉 王, 嫱 姚, 思伟 朱

**Affiliations:** 1 300121 天津，天津市人民医院肿瘤科 Department of Medical Oncology, Tianjin People's Hospital, Tianjin 300121, China; 2 300121 天津，天津市人民医院胸外科 Department of Toracic Surgery, Tianjin People's Hospital, Tianjin 300121, China

**Keywords:** 肺肿瘤, 环境, 烟草, 系统评价, Lung neoplasms, Environment, Tobacco, Systematic review

## Abstract

**背景与目的:**

已有的研究表明：工作环境烟草暴露与非吸烟人群肺癌发生有密切关系并不十分明确，本研究旨在探讨工作环境烟草暴露与非吸烟人群肺癌发生危险的关系。

**方法:**

通过计算机检索Medline（1954年-2010年8月）、CENTRL（the Cochrane central register of controlledtrials）（2010 issue3）、EMBASE（1970年-2010年8月）中国生物医学文献数据库系统（CBM）（1978年-2010年8月）、中国期刊全文数据库（CNKI）（1979年-2010年8月）、中文科技期刊全文数据库（VIP）（1989年-2010年8月）等数据库，收集国内外公开发表的关于工作环境烟草暴露与非吸烟人群肺癌发生关系的研究文献，应用统计软件Stata 11.0进行数据分析，计算其合并优势比（odds ratio, OR）和95%置信区间（confdence interval, CI）。采用*Begg*法对发表偏倚进行量化检测。

**结果:**

最终纳入分析的文章共有22篇，合并分析结果表明工作环境烟草暴露使非吸烟人群肺癌发生率增加了25%（OR=1.25, 95%CI: 1.13-1.39, *P* < 0.001），使非吸烟女性肺癌的发生率增加了22%（OR=1.22, 95%CI: 1.05-1.42, *P*=0.011）；工作环境烟草暴露导致非吸烟男性肺癌的发生率增加了54%，但无统计学意义（OR=1.54, 95%CI: 0.74-3.18, *P*=0.247）。

**结论:**

工作环境烟草暴露是非吸烟人群肺癌发生的一个危险因素，非吸烟女性工作环境的烟草暴露与肺癌的发生关系密切。

## Abstract (en)

**Background and objective:**

It has been reported that there was a close relationship between lung cancer risk and environmental tobacco smoke at workplace. The aim of this study is to explore the relationship between workplace environmental tobacco smoke exposure and lung cancer risk among non-smoking subjects.

**Methods:**

By searching Medline, CENTRL (the Cochrane central register of controlledtrials), EMBASE, CBM, CNKI and VIP *et al*, we collected both domestic and overseas published documents on workplace environmental tobacco smoke exposure and lung cancer risk. Random or fxed effect models were applied to conduct systematic review on the study results, the combined odds ratio (OR) and the 95% confdence interval (CI) were calculated as well.

**Results:**

22 reports were included into the combined analysis, which indicated that 25% lung cancer risk was increased by exposing to workplace environment tobacco smoke (OR=1.25, 95%CI: 1.13-1.39, *P* < 0.001). For female the increased risk was 22% (OR=1.22, 95%CI: 1.05-1.42, *P*=0.011). For male the increased risk was 54%, but it does not reach the statistical signifcance (OR=1.54, 95%CI: 0.74-3.18, *P*=0.247).

**Conclusion:**

Workplace environmental tobacco smoke exposure is an important risk factor of lung cancer risk among non-smoking subjects. Especially for non-smoking women who expose to workplace environment tobacco smoke have a close relationship with lung cancer.

全世界每年大约有超过一百万人死于肺癌，它的死亡人数超过了乳腺癌、前列腺癌和结肠癌的总和^[1]^，大约有90%的肺癌都和烟草的暴露有关^[2]^。肺癌死亡率高，病因复杂。工作环境烟草暴露与非吸烟人群肺癌发生关系是目前研究的焦点之一，但研究结果^[3-5]^并不完全一致。为此选择工作环境烟草暴露与非吸烟人群肺癌发生关系公开发表的资料进行研究，探讨工作环境烟草暴露是否为非吸烟人群肺癌发生的危险因素。

## 1 资料与方法

### 1.1 文献检索

通过计算机检索Medline（1954年-2010年8月）、CENTRL（the Cochrane central register of controlledtrials）（2010 issue3）、EMBASE（1970年-2010年8月）中国生物医学文献数据库系统（CBM）（1978年-2010年8月）、中国期刊全文数据库（CNKI）（1979年-2010年8月）、中文科技期刊全文数据库（VIP）（1989年-2010年8月）等数据库，收集国内外公开发表的关于工作环境烟草暴露与非吸烟人群肺癌发生危险的队列研究或病例-对照研究，检索语种为英语和汉语。以“Lung neoplasm/lung cancer[MeSH] AND Passive smoking/Environmental tobacco smoke [MeSH]”检索Medline、CENTRAL、EMBASE等英文数据库；以“（肺癌/肺肿瘤）AND被动吸烟/环境烟草暴露”检索CBM、CNKI等中文数据库。为尽量避免漏查文献对入选文献的参考文献进行二次检索，相关综述、会议、摘要文章均被检索以发现可能合格的文献。同时辅以手工检索相关期刊，并用Google scholar搜索引擎在互联网上查找相关文献。

### 1.2 纳入研究的筛选

#### 1.2.1 纳入标准

① 研究设计：队列研究或病例-对照研究；②研究对象：病理证实或临床诊断为肺癌的非吸烟患者；③研究方法：各文献研究假设及研究方法相似；④原始数据：原文献提供OR和95%CI或原始数据能够进行OR值及95%CI的计算；⑤划分标准：暴露及各因素分层划分标准基本相似。

#### 1.2.2 排除标准

① 文献质量较差（Quality score, QS < 30%）；②未能提供OR和95%CI或原始数据不能用以计算OR值及95%CI者；③非队列研究或非病例-对照研究。

### 1.3 纳入研究质量评价

采用双人平行评价的方法，按照“观察性流行病学研究报告规范（STROBE）——病例对照研究”所提供的22条标准对每一篇纳入研究文献的质量进行量化评价（表 1）。对于STROBE中的每一条标准，如果入选文献中明确满足则为2分；部分满足为1分；含糊不清或未提及者则为0分。如果某条标准不适用于该研究，则该条标准不记入评分范围。如22条标准全部满足则为44分并以百分数计算（0-100%），高分值的文献质量相对较高。

**1:** 纳入研究16篇文献的质量评价（STROBE声明） STROBE statement-checklist criteria included in 16 reports of passive smoking and lung cancer risk

Items	Recommendation	Number of stydy [*n* (%)]
Title and abstract	1.1 Indicate the study’s design with a commonly used term in the title or the abstract	6 (27.3%)
	1.2 Provide in the abstract an informative and balanced summary of what was done and what was found	16 (72.7%)
Introduction		
Background/rationale	2 Explain the scientific background and rationale for the investigation being reported	17 (77.3%)
Objectives	3 State specific objectives, including any prespecified hypotheses	18 (81.8%)
Methods		
Study desin	4 Present key elemens of study design early in the paper	15 (68.2%)
Setting	5 Describe the setting, locations, and relevant dates, including periods of recruitment, exposure, follow-up, and data collection	20 (90.9%)
Participants	6.1 Give the eligibility criteria, and the sources and methods of ascertainment, and control selection. Give the rationale for the choice of cases and controls	11 (50.0%)
	6.2 For matched studies, give matching criteria and the number of controls per case	10 (45.5%)
Variables	7 Clearly define all outcomes, exposures, predictors, potential confounders, and effect modifiers. Give diagnostic criteria, if applicable	10 (45.5%)
Datasources/measurement	8 For each variable of interest, give sources of data and details of methods of assessment (measurement). Describe comparability of assessment methods if there is more than one group	12 (54.5%)
Bias	9 Describe any efforts to address potential sources of bias	3 (13.6%)
Study size	10 Explain how the study size was arrived at	0 (0)
Quantitative variables	11 Explain how quantitative variables were handled in the analyses. If applicable, describe which groupings were chosen and why	11 (50.0%)
Statistical methods	12.1 Describe all statistical methods, including those used to control for confounding	16 (72.7%)
	12.2 Describe any methods used to examine subgroups and interactions	14 (63.6%)
	12.3 Explain how missing data were addressed	9 (40.9%)
	12.4 If applicable, explain how matching of cases and controls was addressed 12.5 Describe any sensitivity analyses	11 (50.0%)
Results		
Participants	13.1 Report numbers of individuals at each stage of study (eg numbers potentially eligible, examined for eligibility, confirmed eligible, included in the study, completing follow-up, and analysed)	16 (72.7%)
	13.2 Give reasons for non-participation at each stage	8 (36.4%)
	13.3 Consider use of a flow diagram	2 (9.1%)
Descriptive data	14.1 Give characteristics of study participants (eg demographic, clinical, social) and information on exposures and potential confounders	13 (59.1)
	14.2 Indicate number of participants with missing data for each variable of interest	9 (40.9%)
Outcome data	15 Report numbers in each exposure category, or summary measures of exposure	17 (77.3%)
	16.1 Give unadjusted estimates and, if applicable, confounder-adjusted estimates and their precision (eg 95% confidence interval). Make clear which confounders were adjusted for and why they were included	12 (54.5%)
	16.2 Report category boundaries when continuous variables were categorized	14 (63.6%)
	16.3 If relevant, consider translating estimates of relative risk into absolute risk for a meaningful time period	6 (27.3%)
Other analyses	17 Report other analyses done (eg analyses of subgroups and interactions, and sensitivity analyses)	7 (31.8%)
Discusion		
Key results	18 Summarise key results with reference to study objectives	22 (100%)
Limitations	19 Discuss limitations of the study, taking into account sources of potential bias or imprecision. Discuss both direction and magnitude of any potential bias	12 (54.5%)
Interpretation	20 Give a cautious overall interpretation of results considering objectives, limitations, multiplicity of analyses, results from similar studies, and other relevant evidence	11 (50.0%)
Generalisability	21 Discuss the generalisability (external validity) of the study results	7 (12.7%)
Other information		
Funding	22 Give the source of funding and the role of the funders for the present study and, if applicable, for the original study on which the present article is based	9 (40.9%)

### 1.4 资料提取

采用双人平行摘录方法，由两位研究者独立阅读所获文献题目和摘要，在排除明显不符合纳入标准的试验后，对可能符合纳入标准的试验阅读全文以最终确定是否符合纳入标准。两位评价者交叉核对纳入试验的结果，对有分歧而难以确定是否纳入的试验通过讨论或由第三位评价者决定其是否纳入。提取内容包括：①一般资料：题目、作者姓名、发表日期、文献来源；②研究特征：研究对象的一般情况、各组病人的基线可比性；③结局指标：肺癌发生的优势比OR。

### 1.5 统计分析

阅读文献，按照系统评价的要求整理数据，建立数据库并核校数据，对数据进行定量合成。本研究对肺癌发生的流行病学资料采用肺癌发生的优势比OR为效应指标并以95%可信区间（95%CI）表示。双侧*P* < 0.05为有统计学意义。统计学异质性采用*Q*统计量的*I^2^*检验来分析。双侧*P* > 0.05认为各研究间不存在明显的异质性，采用固定效应模型（fixed effect model）合并数据；如果各研究间存在明显的异质性（*P* < 0.05），分析其异质性的来源，对可能导致异质性的因素进行亚组分析。若两个研究组之间存在统计学异质性而无临床或方法学异质性或差异无统计学意义时，采用随机效应模型（random effect model）合并数据。如果各研究间异质性过大则不适合定量合成数据转而采用描述性分析。采用*Begg*法对发表偏倚进行量化检测。统计应用Stata 11.0统计软件完成。

## 2 结果

### 2.1 文献检索结果

初检文献714篇，阅读标题、摘要后排除不符合要求文献636篇，阅读全文后排除文献56篇，最终纳入*meta*分析文献22篇^[3-24]^（表 2），共入组10, 486例受试者，其中病例组4, 192例，对照组6, 294例。文献的研究年限为1971年-1998年。

**2:** 纳入研究的基本特征 General characteristics of included trials

Reference	No. of patients	Gender	QS%	OR	95%CI	Time period	Year of publication	Location
Kabat^[3]^	53	Female	65	0.7	0.3-1.5	1971-1980	1984	USA
Garfinkel^[4]^	76	Female	62	0.9	0.7-1.2	1971-1981	1985	USA
Wu^[5]^	29	Female	67	1.3	0.3-3.3	1981-1982	1985	USA
Lee^[6]^	15	Female	72	0.6	0.2-2.3	1979-1982	1986	England
Shimizu^[7]^	90	Female	66	1.2	0.6-2.6	1982-1985	1988	Japan
Kalandidi^[8]^	89	Female	70	1.4	0.8-2.5	1987-1989	1990	Greece
Wu-Williams^[9]^	415	Female	71	1.2	0.9-1.6	1985-1987	1990	China
Kabat^[10]^	58	Female	65	1.2	0.6-2.1	1983-1990	1995	USA
Reynolds^[11]^	528	Female	68	1.6	1.2-2.0	1986-1990	1996	USA
Sun^[12]^	230	Female	71	1.4	0.9-2.0	Not report	1994	China
Wang^[13]^	135	Female	62	0.9	0.5-1.8	1992-1994	1994	China
Zaridze^[14]^	189	Female	75	0.9	0.6-1.4	Not report	1998	Russia
Zong^[15]^	504	Female	72	1.7	1.3-2.3	1992-1994	1999	China
Lee^[16]^	268	Female	73	1.5	0.5-2.4	1992-1998	2000	Taiwan
Johnson^[17]^	71	Female	71	1.3	0.4-4.0	1994-1997	2001	Canada
Kabat^[3]^	25	Male	65	3.3	1.0-10.6	1971-1980	1984	USA
Lee^[6]^	10	Male	72	1.6	0.4-6.6	1979-1982	1986	England
Kabat^[18]^	41	Male	76	1.0	0.5-2.1	1983-1990	1995	USA
Schwartz^[19]^	257	Male/Female	77	1.5	1.0-2.2	1984-1987	1996	USA
Boffetta^[20]^	650	Male/Female	71	1.2	0.9-1.5	1988-1994	1998	Europe
Boffetta^[21]^	70	Male/Female	63	1.5	0.8-3.0	1994-1996	1999	Europe
Rapiti^[22]^	58	Male/Female	64	1.1	0.3-4.1	1991-1992	1999	India
Kreuzer^[23]^	123	Male/Female	77	1.1	0.7-1.7	1990-1996	2000	Germany
Wang^[24]^	233	Male/Female	70	1.6	0.7-3.3	1994-1998	2000	China

### 2.2 纳入研究的基本特征

最终纳入研究的文献共22篇均为英文文献。根据“流行病学研究报告规范（STROBE）——病例对照研究”对入选的文献进行方法学质量评价，22篇文献的平均得分为69.5 %（62%-77%）。

### 2.3 各独立研究结果的异质性检验

纳入研究的22篇文献以肺癌发生的优势比OR为效应指标，采用*I^2^*统计量进行异质性检验，*I^2^*=12.5% < 50%，*P*=0.288 < 0.05，认为各独立研究结果之间不存在明显的异质性，合并分析均采用固定效应模型。

### 2.4 数据定量合成

纳入研究的22篇文献均提供了较为完整的资料可计算OR和95%CI。定量合并数据后的结果显示，工作环境烟草暴露者患肺癌的风险是未暴露者的1.25倍（OR=1.25, 95%CI: 1.13-1.39, *P* < 0.001）（图 1）。按性别分层分析非吸烟女性患肺癌风险与工作环境烟草暴露的关系，纳入研究的文献有15篇^[3-17]^，分析结果显示工作环境烟草暴露是非吸烟女性肺癌发生的危险因素，暴露者患肺癌的风险是未暴露者的1.22倍（OR=1.22, 95%CI: 1.05-1.42, *P*=0.011）；分析非吸烟男性患肺癌风险与工作环境烟草暴露的关系，纳入研究的文献有3篇^[3, 6, 18]^，分析结果显示暴露者患肺癌的风险是未暴露者的1.54倍（OR=1.54, 95%CI: 0.74-3.18, *P*=0.247），但差别无统计学意义。

**1:**
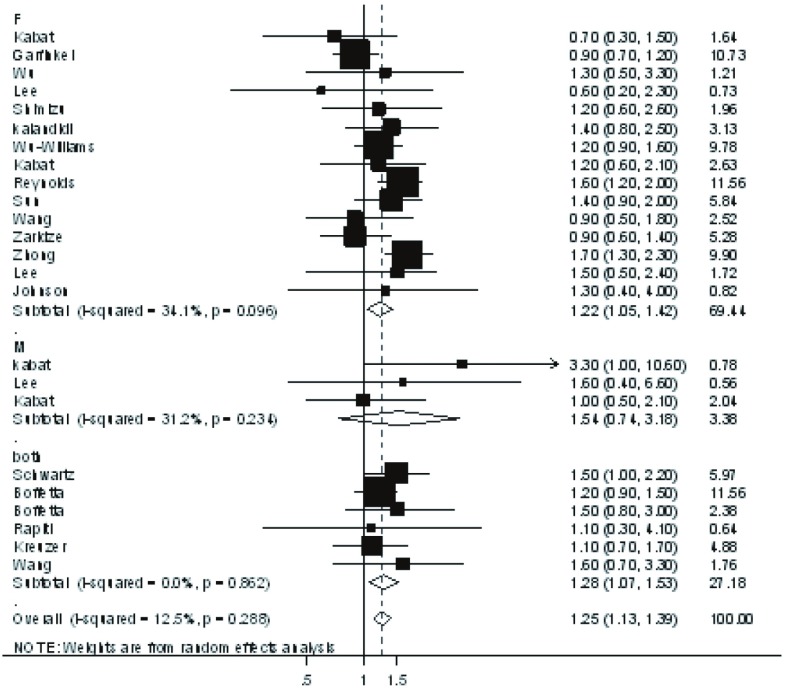
工作环境烟草暴露与非吸烟人群肺癌发生危险的森林图 Forest plot of workplace environmental tobacco smoke exposure and lung cancer risk

### 2.5 发表性偏倚的识别及敏感性分析

*Begg*法量化检测发表偏倚，*Begg'*test中Pr > |z|=0.823 > 0.05，图中各点沿中间水平线均匀分布，且均位于预计95%置信区间内（图 2）；对纳入研究的22篇文献进行敏感性分析，剔除任意一篇文献后*meta*分析OR的95%CI均位于1.007-1.306之间，不包括1在内，剔除前后结论的性质未发生改变（图 3）。

**2:**
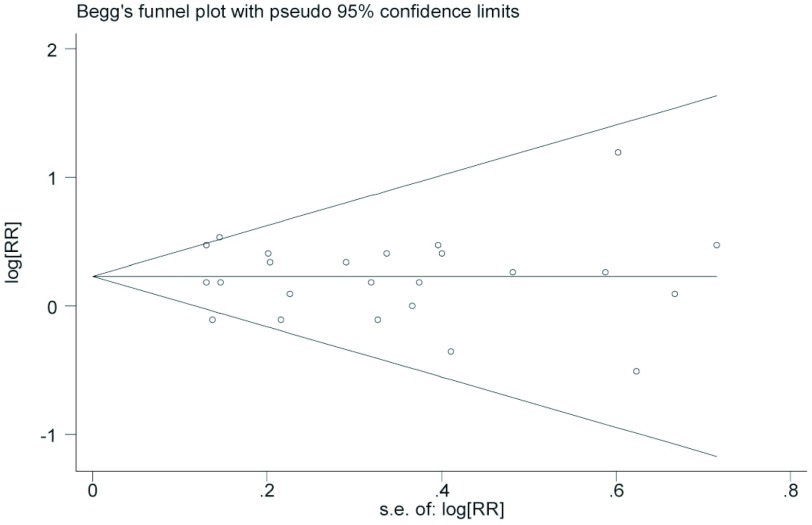
工作环境烟草暴露与肺癌发生危险的漏斗图 The funne plot for workplace environmetal tobacco smoke and lung cancer risk

**3:**
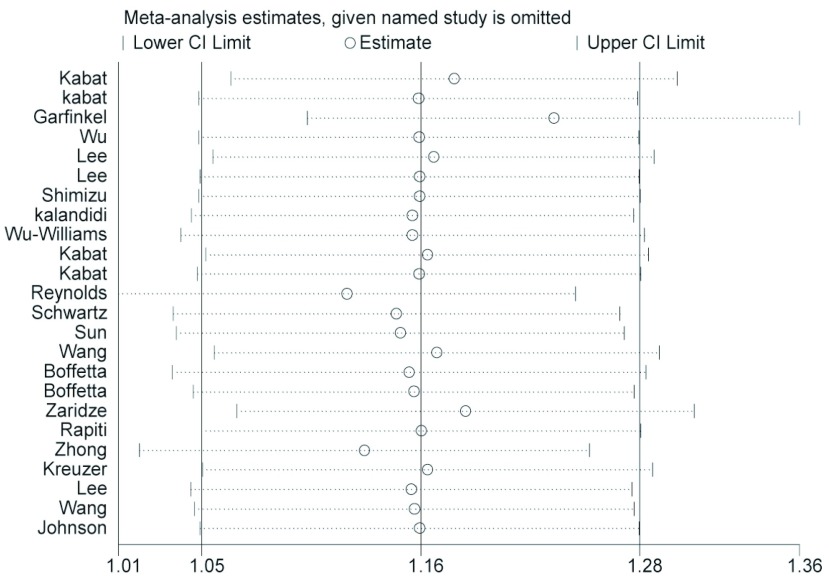
各篇文献对*meta*分析结果的影响 The influence of each trial for the outcome of the systematic review

## 3 讨论

环境烟草烟雾是从燃烧的烟草释放出的侧烟流和吸烟者呼出的烟雾所构成，二者分别占80%和20%。吸烟者吸入的烟雾称之为主流烟雾。环境烟草烟雾的其它成分包括在喷烟时从燃烧的烟头逸出的烟雾和通过卷烟纸所弥散出的气体成分。这些成分被周围空气所稀释，一旦被吸入，尤其是被非吸烟者吸入时，就称之为环境烟草暴露。环境烟草暴露可能导致肺癌的基本原理与吸烟相似，环境烟草烟雾中含有各种有毒物质，包括诱变剂和致癌原如亚硝酸、4-氨基联笨、苯丙芘等。这些有害化学物质在环境烟草烟雾中比在烟草燃烧释放的主流烟雾中还要高。

非吸烟者肺癌在流行病学、危险因素及预后方面有其独特的生物学特征，已经成为肺癌独立的亚型受到越来越多的关注^[25]^。既往的一些*meta*分析结果表明^[26-28]^，环境烟草暴露与非吸烟者和肺癌的发生有一定的关系。本研究发现工作环境烟草暴露使非吸烟人群肺癌发生率增加了25%（OR=1.25, 95%CI: 1.13-1.39, *P* < 0.001），使非吸烟女性肺癌的发生率增加了22%（OR=1.22, 95%CI: 1.05-1.42, *P*=0.011），但与非吸烟男性肺癌的发生关系无统计学意义（*P*=0.247）。研究结论提示女性非吸烟者可能是工作环境烟草暴露导致肺癌的优势人群。

该研究共纳入22篇文献，质量相对较高，且均为英文文献，每篇文献所在杂志的影响因子均在1以上。根据随“流行病学研究报告规范（STROBE）——病例对照研究”制定的标准对每篇文献进行了评分，平均得分为69.5%（62%-77%）；敏感性分析显示剔除任意一篇文献后*meta*分析OR的95%CI均位于1.007-1.306之间，不包括1在内，剔除前后结论的性质未发生改变。对于发表偏倚的评估认为该项系统评价不存在明显的发表偏倚。综上，该项系统评价的原始数据较为真实可靠，*Q*统计量的*I^2^*检验显示各研究不存在明显的统计学异质性，*Begg*法显示不存在发表偏倚。基于上述分析，该研究的结论应较为稳定。

该系统评价也存在一定的缺陷，各研究之间病例组和对照组人群的基线情况不可能完全一致，因此在病例的选择上可能存在一定的偏倚，这种偏倚可能是潜在临床异质性的重要来源；对于工作环境烟草暴露水平的判断主要是通过被调查人员进行描述和回忆，往往受主观因素的影响较为严重，这种差异可能是回忆性偏倚和方法学异质性的一个重要来源。
